# Associations of stigma, loneliness, and treatment self-regulation with HIV medication adherence among individuals with substance use disorder using a mobile health application

**DOI:** 10.3389/fpubh.2024.1440807

**Published:** 2024-11-05

**Authors:** Adati Tarfa, Tarfa Verinumbe, Fan (Ellie) Yang, Olayinka O. Shiyanbola, Cameron Liebert, Sarah Dietz, Rebecca Miller, Ryan P. Westergaard

**Affiliations:** ^1^Section of Infectious Diseases, Department of Internal Medicine, Yale School of Medicine, New Haven, CT, United States; ^2^Division of Social and Administrative Sciences, School of Pharmacy, University of Wisconsin-Madison, Madison, WI, United States; ^3^Division of Infectious Diseases, Johns Hopkins University School of Medicine, Baltimore, MD, United States; ^4^School of Communication, Illinois State University, Normal, IL, United States; ^5^Department of Medicine, School of Medicine and Public Health, University of Wisconsin-Madison, Madison, WI, United States; ^6^School of Medicine and Public Health, University of Wisconsin, Madison, WI, United States

**Keywords:** HIV, stigma, social isolation, substance use disorder, adherence, mobile health, digital health, antiretroviral therapy

## Abstract

**Introduction:**

Medication adherence contributes to poor HIV outcomes, especially among people with HIV and Substance use disorder (SUD). Mobile health applications have been leveraged to improve behavioral health outcomes among this population. Our cross-sectional study examined the relationship between medication adherence with factors such as treatment self-regulation, isolation, and internalized stigma, among people with HIV and SUD using the Addiction Comprehensive Health Enhancement Support System (A-CHESS) mobile app.

**Methods:**

A sample of 208 participants using A-CHESS to improve treatment adherence completed a survey. Adherence was measured using the Four-item Morisky Medication Adherence Scale and dichotomized (maximum score of 20 points considered as adherent). Positive and negative affect was measured separately using Positive Affect Negative Affect Schedule and loneliness was measured using UCLA three-item Loneliness Scale. Internalized stigma was measured using Internalized AIDS-Related Stigma Scale. Competence/Treatment self-regulation was measured using Treatment Self-regulation Questionnaire. Multivariable logistic regression was used to assess the associations of affect, treatment self-regulation, isolation, and internalized stigma, with adherence to antiretroviral therapy adjusting for age, education, and gender.

**Results:**

Among 208 participants in this study, most were Black (*n* = 137; 66%), male (*n* = 156; 75%) and had a mean age of 46 (standard deviation = 11.3). The most reported substances associated with missing HIV medication were alcohol (27%) and cocaine/crack (20%). Logistic regression analysis revealed that internalized stigma was significantly associated with HIV medication adherence (OR = 0.82; 95% CI: 0.70–0.99; *p* = 0.034).

**Conclusion:**

Internalized stigma was significantly associated with HIV medication adherence. Further research is needed to better understand this relationship and develop interventions addressing stigma in people with HIV and SUD.

## 1 Introduction

Antiretroviral therapy (ART) has transformed the health and quality of life of people living with HIV (PWH), enabling them to achieve life expectancies comparable to those without the virus (1). However, the effectiveness of ART depends on appropriate medication adherence (2), which entails following the prescribed timing, dosage, and frequency over the recommended treatment duration (3). Optimal adherence to ART decreases viral load (4), improves immune function (5), reduces mortality rates (6), and enhances the overall quality of life in PWH (7–9).

According to HIV surveillance data from the Centers for Disease Control and Prevention (CDC), only 62.7% of PWH achieve viral suppression (10), indicating suboptimal medication adherence. Prior research indicates that ART adherence is impacted by several complex psychosocial factors, including substance use disorder (SUD) (11, 12). The syndemic theory suggests that multiple co-occurring psychosocial comorbidities interact synergistically to contribute to poor health outcomes for PWH (13). Substance use has been identified as an independent risk factor for poor HIV outcomes within the syndemic context (14, 15). For instance, a prospective study found that individuals using drugs, regardless of their usage pattern (e.g., intermittent use, persistent use), had higher odds of having an unsuppressed viral load than non-users (16). Moreover, increased alcohol consumption has been linked to lower odds of improving ART adherence (17, 18). Therefore, it is imperative to investigate medication adherence within this subpopulation to understand the factors influencing adherence and develop targeted interventions.

Factors impacting care among PWH and SUD operate on multiple levels, including intrapersonal, interpersonal, community, societal, and structural (19). Multiple behavioral change theories explore the factors influencing adherence in chronic disease management (20, 21). Specifically, psychological theories such as the Health Belief Model (22), Social-Cognitive Theory (23), and Theory of Reasoned Action (24) have challenged the passive role of patients in healthcare, emphasizing that adherence depends on individuals being informed, motivated, and convinced of treatment benefits (25, 26). Despite the promises of these theories in understanding medication adherence, they do not account for external factors, such as social relationships, and ones' perception of their relationship which impacts the individual's engagement in a behavior (26).

Additionally, there are still limitations in applying theory-driven evidence-based models to develop effective interventions to understand and address medication adherence (27). A recent scoping review of qualitative studies exploring the complexity of medication adherence in patients with chronic disease found that only 17 studies had a behavior theory-based approach to understanding medication adherence (28). A review of the utility of health behavior theories for developing interventions to promote long-term medication adherence identified that only one study employed an explicit theoretical framework (29). Specifically, in HIV research, many ART adherence interventions have limited or no explicit theoretical basis (30).

Self-determination theory (SDT) is one prominent theory used extensively to promote health behavior change (31, 32). Findings from a recent meta-analysis support the efficacy of SDT interventions in promoting health behavior change (33). SDT suggests that individuals achieve greater self-regulation by internalizing their behaviors, moving from external control to autonomous regulation, with motivation types and environmental factors playing a role in this process (34). A qualitative study of PWH in New York integrated Critical Race Theory, Harm Reduction, and SDT to identify and understand the complex and multi-level factors that drive poor engagement in HIV care focused on the positive impact of the SDT construct of social-relatedness and engagement in care (35). Similarly, SDT was applied to address drug-related harm among PWH who use drugs (36). SDT provides valuable insights into the impact of social environments on addiction among individuals with unmet psychosocial needs, such as relatedness (37).

Psychological theories and approaches are valuable in identifying modifiable factors associated with behaviors like medication adherence (38), and they hold particular promise for individuals with SUD who face unique challenges with adherence. Theory-based interventions can significantly improve health outcomes for people with SUD. The proven efficacy of SDT in diverse health behaviors suggests its potential to effectively identify the complex factors that impact treatment adherence among PWH and SUD. Our study aims to apply Self Determination Theory to examine the association between SDT and ART adherence among individuals with SUD, utilizing a secondary analysis of a larger mobile health study focused on identifying risky behaviors impacting treatment adherence in PWH and SUD.

## 2 Materials and methods

### 2.1 Study design

This cross-sectional study assessed associations between medication adherence among PWH and SUD using survey data collected from March 2019 to June 2019. This study is part of a larger project implementing the Addiction-Comprehensive Health Enhancement Support System (A-CHESS) from March 2019 to March 2020 to enhance support and engagement in care for individuals with HIV and SUD. A-CHESS, developed by the Center for Health Enhancement System Studies at the University of Wisconsin-Madison, offers a comprehensive range of services to address the multifaceted challenges faced by individuals seeking addiction treatment and prevention (39). These services include discussion forums, cognitive behavioral therapy boosters, games, relaxation activities, and educational resources on hepatitis C, HIV, and SUD. Aligned with the components of self-determination theory, A-CHESS aims to enhance self-motivation and wellbeing by catering to the psychological needs of competence, autonomy, and relatedness (40). Our study uses a baseline survey completed by A-CHESS participants.

### 2.2 Participant recruitment

PWH and SUD were identified by research coordinators at HIV clinics in Wisconsin through provider referrals. To be eligible for participation, individuals had to be 18 years or older, have clinical documentation of HIV infection, and have a history of substance use disorder. SUD history was defined by meeting one or more of the following criteria: a positive result on substance-use screening tests, evidence of substance use within the past year, current involvement in a substance-use treatment program, regular participation in a substance-use disorder-oriented support group (at least monthly), or a lifetime history of problematic drug or alcohol use with recent incarceration.

Patient Consent Statement: All participants provided written informed consent before enrollment in the study. The University of Wisconsin-Madison Health Sciences Institutional Review Board (IRB) provided approval for the study.

### 2.3 Data collection and measures

The baseline survey for A-CHESS study participants was administered by trained research staff, who conducted the surveys over the phone or in person. The estimated completion time for the survey was 20–30 min, and all data collected by the research staff were securely stored in a Research Electronic Data Capture (REDCap) database (41). Participants received $50 for completing the baseline survey.

The baseline survey administered to the A-CHESS study participants assessed diverse items related to HIV and substance use such as health service utilization, withdrawal symptoms, and risky sexual behaviors. However, only theory-informed relevant measures were utilized for this study. The selection of specific baseline survey measures was guided by the principles of SDT as outlined in the existing literature.

According to SDT, intrinsic motivation is the satisfaction of the three innate basic psychological needs of autonomy, competence, and relatedness (32). When these needs are satisfied, it is assumed that self-determined forms of motivational regulation guide behavior such as medication adherence (32). Autonomy involves having a sense of volition in determining one's behavior (42). Competence consists of the experience of feeling capable and effective when interacting with one's environment (42, 43). Relatedness involves feeling a sense of support and connection with others (42). Guided by these constructs, we selected positive/negative affect, loneliness scale, internalized AIDS-related stigma, and treatment self-regulation as independent measures.

#### 2.3.1 Dependent variable

Our dependent variable of interest is ART adherence, measured using the Four-item Morisky Medication Adherence Scale (MMAS-4) (44). The MMAS-4 is widely used to capture self-reported medication-taking behavior (45). It consists of four items, including questions such as “How often do you forget to take your HIV medications?” and “If you feel worse from taking your HIV medications, how likely are you to stop taking it?” with response options ranging from 1 (indicating a higher frequency or likelihood) to 5 (indicating a lower frequency or likelihood). Each question was assigned one point, resulting in a total score of 20. Adherence was defined as achieving the maximum score of 20 points, while nonadherence scored as any total points below 20 (46). Previous research has shown that the MMAS-4 effectively evaluates ART adherence (47). MMAS-4 has also demonstrated a moderate to high concordance with electronic medication monitoring devices (48). The sensitivity and specificity of the MMAS were reported as 81% and 44%, respectively. We calculated a Cronbach's alpha of 0.9878 for the medication adherence scale.

#### 2.3.2 Independent variables

##### 2.3.2.1 Positive Affect Negative Affect Schedule (PANAS)

To measure individuals' feelings of self and engagement in personally meaningful activities and their sense of constraint and frustration, we utilized the Positive and Negative Affect Schedule (PANAS) (49). The PANAS has strong reported validity with such measures as general distress and dysfunction, identified as feelings that undermine autonomy (50). Negative affect, on the other hand, involves experiencing the world more negatively (51). Participants were asked to respond to the 20 items of the PANAS, where individuals indicate to what extent they feel 20 emotions, including Interested, Distressed, Excited, Upset, Strong, Guilty, or Scared. The emotions are scored using a 5-point Likert scale from 1 point for “Very slightly or not at all” to 5 points for “Extremely.” The final score is derived from the sum of the 10 items on both the positive and negative sides. The reliability and validity of the PANAS scale are moderately good (49, 50). The Cronbach alpha coefficient for the Positive Affect Scale is 0.87; for the Negative Affect Scale is 0.89. We measured negative and positive affect as two distinct continuous measures ranging from 10 to 50 (52).

##### 2.3.2.2 Internalized AIDS-Related Stigma Scale

Internalized stigma refers to the process whereby individuals living with HIV internalize negative societal attitudes and develop self-deprecating beliefs about themselves. We utilized the Internalized AIDS-Related Stigma Scale to measure internalized AIDS-related stigma on a six-item scale, which asks the following questions: (1) It is difficult to tell people about my HIV infection. (2) Being HIV-positive makes me feel dirty. (3) I feel guilty that I am HIV positive. (4) I am ashamed that I am HIV positive. (5) I sometimes feels worthless because I am HIV positive. (6) I hide my HIV status from others (53). Each item offers a binary (yes/no) response, and the total scale score is computed as the sum of the items.

##### 2.3.2.3 The University of California-Los Angeles 3-item Loneliness Scale

We utilized the three-item Loneliness Scale developed by the University of California-Los Angeles (UCLA) to measure social relatedness (54). Loneliness encompasses isolation, disconnection, and a lack of belongingness (55). The scale assesses three aspects of loneliness: feeling left out, isolated, and lacking companionship. Ranging from hardly ever (or never) to some of the time or often, participants are asked to indicate the frequency with which they experience the following: lack of compassion, feeling left out and feeling isolated from others. We calculated a continuous scale score for the loneliness measure, with scores falling within the range of 3–12 (56). Cronbach's alpha reliability coefficient is 0.79.

##### 2.3.2.4 Treatment Self-regulation Questionnaire (TSRQ)

Competence refers to an individual's experience of feeling capable and effective when interacting with their environment. In the context of SDT, competence is one of the three basic psychological needs that, when satisfied, contribute to intrinsic motivation and wellbeing.

We assessed competence using the Treatment Self-regulation Questionnaire (TSRQ) (57). Treatment self-regulation involves regulating and managing one's own behaviors, thoughts, and emotions related to adhering to a treatment regimen (58). It reflects an individual's perceived competence in effectively engaging in self-directed actions to adhere to a behavior. The scale includes six questions about self-regulation related to substance use, such as: “I want to reduce my use because...” with response options like, “I want to take responsibility for my own health,” “I believe this is the best choice for my health,” and “It aligns with my life goals.” By assessing treatment self-regulation, researchers can gain insights into an individual's perceived competence in managing their health-related behaviors and the extent to which they feel capable of adhering to the treatment requirements. On a 5-point Likert scale, participants were asked to rate the self-regulation questions from “Not True” to “Very True.” Each score on the scale is worth one point, and the total points are 30. We calculated a Cronbach alpha of 0.85, indicating good internal consistency.

##### 2.3.2.5 Sociodemographic information

We asked participants to provide information regarding their gender. We collected age as a continuous variable, allowing participants to enter their exact age. We inquired about the highest level of education completed by each participant and categorized it as either having completed college or not. For participant race, we presented options including White, Black or African American, Asian, Native Hawaiian or other Pacific Islander, and American Indian or Alaskan Native. We also allowed participants to enter a different race through an open text field.

### 2.4 Statistical analysis

Frequency distributions and descriptive statistics were used to summarize the characteristics of A-CHESS participants. All categorical variables are described as numbers and percentages.

Multivariable logistic regression was used to examine the association between the dependent variable, medication adherence, and the primary independent variables of interest (AIDS-related stigma, loneliness, positive/negative affect, treatment self-regulation). Other covariates were selected based on their hypothesized association with the primary covariates of interest and medication adherence. These variables are self-reported and include participants' sociodemographic characteristics. Demographic characteristics reported included age, gender (“male,” “female,” and “transgender”), educational level, and employment status. Odds ratios (OR) and 95% confidence intervals were estimated. A two-sided *p* < 0.05 was considered statistically significant. All the statistical analyses will be carried out using IBM SPSS Statistics (Version 28.0).

## 3 Results

### 3.1 Participant characteristics

A total of 208 individuals with HIV and SUD were enrolled in the study and completed the baseline survey. Most participants were male (75%) with a mean age of 46.34 (standard deviation 11.26). Among the participants, 65.9% identified as Black/African American. Additionally, 57.2% reported being single, and ~66.8% were unemployed (Table 1). Table 2 shows the correlation between key variables. Several key variables were significantly correlated. Stigma was negatively correlated with medication adherence (*r* = −0.17, *p* < 0.05). Additionally, loneliness was positively correlated with negative affect (*r* = 0.48, *p* < 0.001) and negatively correlated with positive affect (*r* = −0.31, *p* < 0.001).

**Table 1:** Sociodemographic characteristics of A-CHESS participants at baseline.

**Socio-demographic characteristics^a^**	**Frequency (%) *n* = 208**	**Mean (SD)**
**Gender**		
Male	156 (75.0)	
Female	48 (23.1)	
Other	4 (1.9)	
**Age (years)**		46.3 (11.3)
50–59	74 (35.6)	
40–49	49 (23.6)	
30–39	38 (18.3)	
18–29	23 (11.1)	
60+	23 (11.1)	
**Race**		
Black/African American	137 (65.9)	
White	64 (30.8)	
Other	8 (3.9)	
American Indian/Alaskan Native	5 (2.4)	
**Relationship status**		
Single	119 (57.2)	
Partnered	89 (42.8)	
**Education**		
High school graduate or GED	72 (34.6)	
Some college or 2-year degree	67 (32.2)	
Some high school but did not graduate	49 (23.6)	
More than a 4-year college degree	9 (4.3)	
4-year college graduate	7 (3.4)	
8th grade or less	4 (1.9)	
**Employment status**		
Unemployed	139 (66.8)	
Employed	69 (33.2)	

SD, standard deviation.

^a^Variable distributions are reported as n (%) unless otherwise specified.

**Table 2:** Zero-order correlations for the key variables.

**Key variables**	**1**	**2**	**3**	**4**	**5**	**6**
1. Medication adherence	1.0					
2. Loneliness	−0.13	1.0				
3. Stigma	−0.17^*^	0.24^**^	1.0			
4. Treatment self-regulation	0.02	0.02	−0.11	1.0		
5. Negative affect	−0.10	0.48^***^	0.27^***^	−0.02	1.0	
6. Positive affect	0.10	−0.31^***^	−0.24^***^	0.14^*^	−0.31^***^	1.0

^*^p < 0.05.

^**^p < 0.01.

^***^p < 0.01.

### 3.2 Associations of positive and negative affect, treatment self-regulation, stigma, loneliness with medication adherence

Unadjusted and adjusted logistic regression examined the associations between SDT constructs and medication adherence among individuals with HIV and SUD. Adjusted multivariable logistic regression revealed that stigma was significantly associated with medication adherence (adjusted odds ratio = 0.83, 95% CI (0.70, 0.99), *p* = 0.04]. Higher levels of stigma were associated with reduced odds of medication adherence. However, the analysis did not reveal significant associations between medication adherence and other SDT constructs, including loneliness, negative affect, Treatment Self-Regulation (TSR), and positive affect (Table 3).

**Table 3:** Associations of positive and negative affect, treatment self-regulation, stigma, loneliness with medication adherence.

**Variable**	**Unadjusted OR (95% CI)**	***p*-value^a^**	**Adjusted^a^ OR (95% CI)**	***p*-value^a^**
Age			1.00 (0.97–1.02)	0.76
**Education**				
Less than college				–
College			0.41 (0.22–0.76)	0.005
**Gender**				
Male			–	–
Female			1.38 (0.69–2.77)	0.37
Loneliness	0.95 (0.85–1.06)	0.37	0.96 (0.86–1.08)	0.53
Stigma	0.86 (0.73–1.00)	0.06	0.83 (0.70–0.99)	0.04
Treatment self-regulation	0.97 (0.53–1.79)	0.93	0.91 (0.49–1.71)	0.77
Negative affect	1.00 (0.97–1.03)	0.94	0.99 (0.96–1.02)	0.51
Positive affect	1.00 (0.97–1.05)	0.62	1.00 (0.97–1.04)	0.82

Hosmer and Lemeshow Test showed an acceptable model fit (χ^2^ = 3.52, p = 0.91).

^a^p-value significant at ≤ 0.05 level.

CI, confidence interval; OR, odds ratio.

The overall model, including all variables, was statistically significant [χ^2^ (df = 8, *N* = 208) = 17.19, *p* = 0.03], indicating that the model can distinguish between adherent respondents and those not adherent to ART. The model explained between 8.2% (Cox and Snell *R* square) and 10.9% (Nagelkerke *R* squared) of the variance in adherence and correctly classified 62.9% of cases.

## 4 Discussion

In this study, we assessed the associations of SDT constructs including stigma, positive affect, negative affect, treatment self-regulation, and loneliness with medication adherence among individuals with HIV and SUD. While affect, treatment self-regulation, and loneliness were not significantly associated with medication adherence, stigma was found to be significantly associated with medication adherence. This finding highlights that stigma may contribute to HIV and substance use on treatment adherence, suggesting the need for targeted interventions to address and reduce stigma-related barriers.

The differential findings between stigma and loneliness suggest that it may be important to consider the unique contributions of different factors related to self-determination theory in understanding medication adherence. While both stigma and loneliness are measures of social relatedness, only stigma showed a significant association with medication adherence. These findings suggest that stigma may have a more noticeable impact on medication adherence than feelings of loneliness or lack of social connectedness. Addressing and reducing stigma could potentially play a role in improving medication adherence among individuals with HIV and substance use disorders, and may be considered as a potential factor when designing interventions.

The results of our study align with the existing literature that has consistently associated stigma to poor adherence to ART among individuals with HIV (59–61). However, it is worth noting that few studies have reported the psychosocial mechanisms through which stigma operates, including factors associated with the development of stigma. One study of PWH shows that internalized stigma creates negative appraisal, leading to anxiety and expectations of negative judgment from others, which likely leads to suboptimal medication adherence (62). Another study showed that a person's perceived severity of stigmatizing attitudes that exist in their community leads to internalized stigma, which lowers medication adherence (63). A review of 38 studies reporting either cross-sectional or prospective analyses of the association of HIV-related stigma to medication adherence found substantial empirical evidence linking stigma to adherence. However, these studies and ours did not explore the multiple domains of stigma (64). These findings call for further research to explore the underlying processes through which stigma influences medication adherence, allowing for more targeted interventions to address stigma-related barriers.

Our study focused solely on internalized AIDS-related stigma and did not investigate the impact of intersectional stigmas. Individuals with HIV and substance use disorders may face multiple stigmatized identities (related to their HIV and SUD diagnosis) that can impact their health-seeking behaviors (65). Future studies should examine the effects of various stigmas, such as HIV-related stigma and substance use disorder-related stigma, to understand their influence on medication adherence.

Our findings shed light on the potential role of autonomy in medication adherence. Although autonomy measures, such as affect, were included in our study, they did not show a significant association with medication adherence. Our findings contrast with previous studies that have reported associations between negative affect and medication adherence (66–68). It is vital to differentiate negative affect from depression and consider the conceptual and theoretical differences between these constructs (69). Our findings differ from studies showing unique positive associations between positive affect and health-related outcomes, including viral load suppression in those living with HIV (70). One study showed that increased positive states of mind mediated the association between perceived social support and improved ART adherence (71). In a study of PWH who use methamphetamine, positive affect was independently associated with ART adherence (15).

Studies examining competence as a contributing factor in medication adherence within the framework of self-determination have been relatively limited. Our study aimed to address this gap by investigating competence in the context of treatment self-regulation related to substance use. While previous research has highlighted the importance of perceived competence as a strong predictor of adherence, these studies have primarily focused on competence in general without explicitly considering its relationship with substance use (72). Our study did not find clear evidence that competence in managing SUD is associated with ART adherence.

Although theoretical frameworks, such as self-determination theory, provide a systematic approach to organizing psychosocial factors and developing tailored intervention messages and strategies for promoting behavioral changes (73), it is important to acknowledge that medication adherence is a complex behavior influenced by various factors beyond a single theory. External factors can significantly impact medication adherence, as such, there may be a need to consider multiple behavioral theories in understanding this phenomenon (29).

While our study focused on self-determination theory as a framework for examining autonomy, competence, and relatedness in relation to medication adherence, other behavioral theories may offer additional insights. Exploring the contributions of alternative theories that account for HIV and SUD, such as Capability, Opportunity, and Motivation–Behavior (74), could provide a more comprehensive understanding of medication adherence in individuals with HIV and substance use disorders. However, to facilitate meaningful comparisons and enhance the overall evidence base, it is crucial to ensure consistency in the operationalization of theories across studies (75).

Our study has certain limitations that need to be acknowledged. First, the cross-sectional nature of the survey prevents us from establishing causality between socio-behavioral factors and medication adherence. While the Morisky Four-item medication adherence scale provides reasonable estimates of medication-taking behavior, it may not serve as an adequate explanatory tool for understanding why patients are not adherent, potentially leading to a weak relationship between the Morisky scale and objective clinical outcome measures (76). The loneliness scale was used as a proxy for social relatedness. Still, it may not fully capture the extent of one's relationship with others and only accounts for one's perception of their relationships. Therefore, this study does not include measurements related to social interaction, including the frequency and intensity of the interactions, which can be beneficial for understanding medication-taking behaviors. Additionally, reliance on self-report recall for measuring adherence, while validated in previous research (77), is susceptible to recall bias. Although patient self-report is one of the critical indirect methods of measuring medication adherence (76), our study could benefit from improved data collection methods, such as integrating innovative technologies like smart pill bottles or medication event monitoring systems, to gather more frequent and objective data on medication adherence.

## 5 Conclusion

Our study highlights the association between internalized stigma and medication adherence among individuals with HIV and SUD. The findings suggest that interventions focused on medication adherence may benefit from considering the role of stigma when examining medication-taking behaviors. Future research should explore how stigma influences medication adherence and explore the impact of intersectional stigmas for both HIV and substance use. Addressing medication adherence challenges may require a multifaceted and interdisciplinary approach that considers the complex interplay of individual, social, and systemic factors.

## Data Availability

The raw data supporting the conclusions of this article will be made available by the authors, without undue reservation.
